# Editorial: Patent Foramen Ovale (PFO) Closure for Prevention of Stroke

**DOI:** 10.3389/fneur.2021.718457

**Published:** 2021-06-28

**Authors:** Damianos G. Kokkinidis, Aristeidis H. Katsanos, George Giannakoulas, Harsimran S. Singh, Guillaume Turc, Vincent Thijs

**Affiliations:** ^1^Section of Cardiovascular Medicine, Yale University School of Medicine, Yale New Haven Hospital, New Haven, CT, United States; ^2^Division of Neurology, McMaster University/Population Health Research Institute, Hamilton, ON, Canada; ^3^Department of Cardiology, AHEPA University Hospital, Thessaloniki, Greece; ^4^Division of Cardiology, Department of Medicine, Weill Cornell Medicine-New York Presbyterian Hospital, New York, NY, United States; ^5^Department of Neurology, GHU Paris Psychiatrie et Neurosciences, Université de Paris, INSERM U1266, and FHU Neurovasc, Paris, France; ^6^Stroke Theme, Florey Institute of Neuroscience, Melbourne, VIC, Australia

**Keywords:** patent foramen ovale, stroke, cryptogenic stroke, PFO closure device, stroke prevention

Stroke is the second leading cause of death worldwide and fifth leading cause of death in the United States (1). The term cryptogenic strokes is used to define strokes for which a cause cannot be identified and account for almost 40% of all the ischemic strokes. Patent foramen ovale (PFO) can potentially explain some of those strokes since it allows right-to-left shunting and was found to be more common in patients with cryptogenic strokes (40%) vs. the general population (25%). After the long-term results of the RESPECT trial and the publication of Gore REDUCE and CLOSE trials and multiple meta-analyses showing benefit from PFO closure in patients with history of cryptogenic stroke, PFO closure has regained a lot of popularity but is also attracting criticism when performed in patients with borderline indications (2–9). Our aim with this Research Topic was to collect a number of well-conducted primary studies, meta-analyses or state of the art narrative reviews on different questions and controversies regarding PFOs role in cryptogenic strokes. In this editorial, we present and put in context compared to the existing literature, the highlights of the studies of this Research Topic.

But what is the real prevalence of PFO? Koutroulou et al. conducted a systematic review of studies investigating the PFO rates according to different diagnostic imaging modalities. They found significant heterogeneity with prevalence rates ranging from 24.2% in autopsy studies to 23.7% in studies using transesophageal echocardiogram for the diagnosis vs. 31.3% in studies using transcranial doppler and only 14.7% in studies using only transthoracic echocardiogram. As expected, PFO prevalence was higher among patients with prior cerebrovascular events vs. those without prior cerebrovascular events, across all different diagnostic modalities and the autopsy series.

However, whether PFO (co)existence is the direct cause of stroke in patients with cryptogenic ischemic stroke remains an unanswered question. Ioannidis and Mitsias, in their state-of-the-art review, argue that PFOs can act as the direct cause vs. risk factor, or an even incidental finding in some patients with cryptogenic stroke. They provide an overview of the potential stroke mechanisms including paradoxical embolism, *in situ* clot formation or atrial tachyarrhythmias in the setting of a hypermobile atrial septum. Risk factors include the size and morphology of the PFO and the degree of the shunt. The authors present and explain the Risk of Paradoxical Embolism (RoPE) score and its use in patients with PFO. Low RoPE scores suggest low probability of pathogenic PFO and relatively higher probability of recurrent stroke events while higher RoPE scores suggest higher probability of pathogenic PFO but lower probability of recurrent events. The first of the mechanisms that Ioannidis and Mitsias proposed and analyzed is paradoxical embolism which originates from concomitant deep vein thrombosis (DVT). It seems that the prevalence of DVT and pulmonary embolism (PE) in those patients is higher than previously thought. Zietz et al. performed a systematic review of the association between DVT/ PE and PFO existence in patients presenting with cryptogenic stroke. They found eight eligible studies in total, with the DVT frequency ranging from 7 to 27% and the PE frequency ranging from 4.4 to 37%. They also examined the reversed association and they found that the presence of PFO in patients with PE was associated with higher rates of ischemic brain lesions. Given those findings, it is probably reasonable to maintain a lower threshold for DVT/PE screening in patients who present with stroke and are subsequently found to have a PFO.

On the other hand, the presence of PFO in the setting of ischemic stroke, was shown to be negatively associated with presence of AF, according to a meta-analysis conducted by Ze-Jun Chen and Thijs. The authors included 14 studies and 13,425 patients comparing AF rates in stroke patients with PFO vs. those without a PFO. They found that patients with a PFO were 48% less likely to have AF compared to those without a PFO. Their results remained significant after performing separate analyses for cross-sectional and longitudinal studies and in different age groups (>60 years old vs. <60 years old). Those findings -although potentially subject to detection bias- support that patients with PFO are not at an increased risk of arrhythmia compared to the general stroke population and may actually have a lower risk. Impaired left atrial (LA) mechanical function has been suggested to be one of the possible causes of cryptogenic strokes, since it can be associated with blood stasis and thrombus formation, while a few studies have even associated impaired LA function with presence of PFO. Speckle tracking is one of the non-invasive methods to evaluate the LA function. Gazagnes et al. studied the association between LA longitudinal strain and presence of PFO in patients who presented with cryptogenic stroke. Interestingly, no association was found, even in the subgroup of patients with PFO and atrial septal aneurysm. Their results were probably limited by their small size and future studies are anticipated.

Collado and Kavinsky discuss the need for a Heart-Brain team approach in PFO closure. The authors wrote a state-of-the-art opinion review presenting the relatively novel concept of the Heart-Brain team. They emphasize that after 2017, PFO closure for stroke preventions in young patients with prior stroke has resurrected and thus in order to avoid under-treatment or overenthusiasm about the invasive options, we should approach those patients with a multi-disciplinary Heart-Brain approach, including neurologists, general cardiologists and interventionalists among others. The Heart-Brain approach can probably provide the best possible consultation, decision making and outcomes for patients with PFO. Multidisciplinary discussion becomes of particular importance especially given the favorable outcomes even in the non-invasive, pharmacological arms of some of the RCTs and large registries on PFO closure. The explanation for this discrepancy might be explained by other non-PFO related risk factors for stroke which are concomitantly present in some of the patients who present with stroke and are found to have a PFO. In order to shed light into this theory, Kahles et al. analyzed data from the International PFO Consortium Study and tried to identify risk factors associated with prior stroke in patients with PFO. Their results were interesting suggesting that both PFO related (right-to-left shunt) and PFO unrelated (hypertension, diabetes, hypercholesterolemia, coronary artery disease, BMI, age) factors were associated with the likelihood of prior stroke and can potentially explain why there is heterogenous benefit among patients who receive a PFO closure device.

The discussion for PFOs role in cryptogenic stroke and the utility of PFO closure for given patient subgroups is still ongoing. We hope that our guest issue provides new insights to the existing literature and creates questions that might be answered in the future.

## Author Contributions

All authors listed have made a substantial, direct and intellectual contribution to the work, and approved it for publication.

## Conflict of Interest

The authors declare that the research was conducted in the absence of any commercial or financial relationships that could be construed as a potential conflict of interest.
